# The C-C Chemokines CCL17 and CCL22 and Their Receptor CCR4 in CNS Autoimmunity

**DOI:** 10.3390/ijms18112306

**Published:** 2017-11-02

**Authors:** Stefanie Scheu, Shafaqat Ali, Christina Ruland, Volker Arolt, Judith Alferink

**Affiliations:** 1 Institute of Medical Microbiology and Hospital Hygiene, University of Düsseldorf, 40225 Düsseldorf, Germany; Stefanie.Scheu@uni-duesseldorf.de (S.S.); Shafaqat.Ali@med.uni-duesseldorf.de (S.A.); 2 Department of Psychiatry, University of Münster, 48149 Münster, Germany; Christina.Ruland@ukmuenster.de (C.R.); Volker.Arolt@ukmuenster.de (V.A.); 3 Cells in Motion, Cluster of Excellence, University of Münster, 48149 Münster, Germany; 4 Department of Psychiatry, Alexian Brothers Hospital, 48149 Münster, Germany

**Keywords:** chemokines, multiple sclerosis, CCR4, CCL17, CCL22, CNS autoimmunity, experimental autoimmune encephalomyelitis, neuroinflammation, migration, dendritic cells

## Abstract

Multiple sclerosis (MS) is a chronic inflammatory demyelinating disease of the central nervous system (CNS). It affects more than two million people worldwide, mainly young adults, and may lead to progressive neurological disability. Chemokines and their receptors have been shown to play critical roles in the pathogenesis of experimental autoimmune encephalomyelitis (EAE), a murine disease model induced by active immunization with myelin proteins or transfer of encephalitogenic CD4^+^ T cells that recapitulates clinical and neuropathological features of MS. Chemokine ligand-receptor interactions orchestrate leukocyte trafficking and influence multiple pathophysiological cellular processes, including antigen presentation and cytokine production by dendritic cells (DCs). The C-C class chemokines 17 (CCL17) and 22 (CCL22) and their C-C chemokine receptor 4 (CCR4) have been shown to play an important role in homeostasis and inflammatory responses. Here, we provide an overview of the involvement of CCR4 and its ligands in CNS autoimmunity. We review key clinical studies of MS together with experimental studies in animals that have demonstrated functional roles of CCR4, CCL17, and CCL22 in EAE pathogenesis. Finally, we discuss the therapeutic potential of newly developed CCR4 antagonists and a humanized anti-CCR4 antibody for treatment of MS.

## 1. Introduction

Multiple sclerosis (MS), a chronic inflammatory disease of the central nervous system (CNS), is characterized by mononuclear cell infiltration, demyelination, and neuronal death [1,2,3]. Experimental autoimmune encephalomyelitis (EAE), a well-established murine model that replicates key pathological features of MS, is induced by active immunization with myelin proteins or transfer of encephalitogenic CD4^+^ T cells [4,5].

A CD4^+^ T-helper (Th)-cell-mediated autoimmune response is an important prerequisite of EAE initiation. Early studies implicated interferon (IFN)-γ producing CD4^+^ Th type 1 (Th1) cells as the major effector cell type in the pathogenesis of EAE [6]. Meanwhile, CD4^+^ Th17 cells, which express various cytokines, including interleukin (IL)-17A, IL-17F, IL-21, and IL-22, have been shown to play a decisive pathogenic role in EAE [7,8]. IL-23, a member of the IL-12 cytokine family, is a key player in Th-17 development and maintenance. It is composed of a p19 subunit and a p40 subunit, the latter being shared with IL-12, and is mainly produced by dendritic cells (DCs) [9]. EAE pathogenesis consists of two phases. First, there is an initial EAE priming phase wherein DCs migrate to peripheral lymph nodes for autoantigen presentation, which is required for the induction of encephalitogenic Th cells. Second, encephalitogenic Th cells are recruited to the CNS to re-encounter the cognate antigen on local and CNS-invading antigen presenting cells [4,10]. These immune-cell trafficking processes, which are integral to CNS immunity, are orchestrated by chemokines, which are small chemotactic polypeptides. Chemokines and their receptors further modulate antigen presentation, cytokine production, effector and memory T-cell differentiation, as well as regulatory T cell (Treg) function [11].

Thus far, some 48 chemokine ligands and more than 20 chemokine receptors have been identified in humans [12]. A number of excellent reviews have described the roles of particular chemokines and their receptors in homeostasis and inflammation [11,13,14]. The aim of this review is to highlight the involvement of a C-C class chemokine/receptor axis, consisting of C-C chemokine ligands 17 (CCL17) and 22 (CCL22), and C-C chemokine receptor 4 (CCR4), in CNS autoimmunity. To this end, we review clinical studies on the expression of CCR4 and its ligands in MS. We highlight recent findings that underscore the functional role of CCL17 and CCR4 in the pathogenesis of EAE and its specific effects on basic DC functions. Finally, the potential of this chemokine/receptor axis as a novel therapeutic target for MS will be discussed.

## 2. The Protein Family of Chemokines and Chemokine Receptors

### 2.1. Structural Characteristics of Chemokines

Chemokines constitute a superfamily of small chemotactic proteins which act as modulators of leukocyte trafficking during homeostasis and inflammatory immune responses [11,15]. Chemokine signaling via chemokine receptors is critical for immune responses in the CNS and has been implicated in a wide range of clinical conditions, including neurodegenerative diseases, CNS autoimmunity, and ischemic injuries [13,16,17,18].

Structurally, chemokines contain conserved cysteine residues, the number and position of which are defining structural characteristics of the four subfamilies (or classes), namely C-X-C, C-C, C, and C-X3-C chemokines [12]. C-X-C chemokines (a.k.a. α-chemokines) have two cysteine residues separated by a single variable amino acid and exert their chemotactic effects on neutrophils, T and B lymphocytes, and natural killer (NK) cells. C-C chemokines (a.k.a. β-chemokines) have two adjacent cysteine residues and can recruit monocytes/macrophages, NK cells, T lymphocytes, basophils, and eosinophils to regions of inflammation. The C chemokines (a.k.a. γ-chemokines), namely lymphotactin α (XCL1) and lymphotactin β (XCL2), contain a single cysteine residue in the conserved position and act on T lymphocytes specifically. Finally, the C-X3-C chemokine (a.k.a. δ-chemokine) family, of which there is only one identified member in humans known as CX3CL1 or fractalkine, is characterized by N-terminal cysteine residues separated by three variable amino acids. CX3CL1 exists in a soluble form as well as a membrane-bound form anchored by a mucin-like domain [19]. Chemokine receptors are generally seven-transmembrane, G protein-coupled receptors [11]. The few exceptions that do not signal through G proteins are referred to as atypical chemokine receptors, or chemokine “decoy” receptors, that may mediate scavenging and other functions [20].

### 2.2. Chemokine Networks

Chemokines and their receptors form complex functional networks: many chemokines bind more than one receptor, and most chemokine receptors bind multiple chemokines. This chemokine/receptor promiscuity may provide functional redundancies [11]. Chemokine receptor activity can also be modulated by dimerization and engagement with other receptors [21]. For example, CXCR4 and CCR5 both heterodimerize and localize together with CD4, selectins, and integrins in the microvilli of various cell types [22,23]. The mechanism and functional relevance of chemokine receptor oligomerization are not well understood, but it has been suggested that it may improve the efficiency of the chemotactic process [21]. Although monomeric chemokine forms have been shown to induce chemotaxis, many chemokines also form dimers readily or build different oligomeric forms that may play yet unexplained roles in the overall process of cell migration [24]. Finally, the expression pattern of chemokines and their receptors by immune cells, which is highly heterogenic, is under spatial and temporal control [11]. These characteristics underlie a high level of complexity for chemokine/chemokine receptor actions. For a detailed review of chemokine dimerization and crosstalk of chemokines, receptors, and associated intracellular signaling pathways, we refer to prior reviews [21,25].

## 3. C-C Chemokine Receptor 4 (CCR4) and Its Ligands C-C Chemokine Ligand 17 (CCL17) and 22 (CCL22) in Homeostasis and Inflammation

### 3.1. Identification and Cellular Expression of CCR4

CCR4 was originally cloned from a human basophilic cell line [26]. The murine orthologue, cloned a year later, was found to be highly expressed in thymus, T cell lines, and peripheral blood T cells [27,28]. It has since been established that CCR4 is highly expressed in functionally distinct T cell subsets, including activated T cells, Th2 cells, and Treg cells. Th1 and Th2 clones (or polarized Th cell subsets) may express different chemokine receptor profiles associated with specific migratory capacities, with Th1 clones preferentially expressing CXCR3 and CCR5, and Th2 cells expressing higher levels of CCR4 and CCR8 [29,30]. Of note, also human and mouse Th17 cells, highly relevant in CNS autoimmune pathology, have been described to express CCR4 [31,32,33]. Specifically, human memory Th17 cells were found to coexpress CCR4 together with CCR6. CCR4 expression has further been determined on platelets, NK cells, monocytes, macrophages, and DCs [26,34,35,36,37,38].

### 3.2. Involvement of CCR4 in Various Diseases

The strong expression of CCR4 on skin-homing T cells expressing cutaneous lymphocyte antigen and on T cells polarized towards the Th2 phenotype is suggestive of CCR4 involvement in skin-associated immune responses and allergies [38,39]. However, *Ccr4* knockout mice showed no apparent phenotype in an experimental Th2-dependent allergic airway inflammation model [35]. It was shown later that CCR4 deficiency mediated enhanced protection against a Th2-type allergic airway disease upon challenge with *Aspergillus conidia* [40]. Furthermore, Th2 cells involved in allergic airway disease models express CCR4, and CCR4^+^ T cells from asthmatic patients are a predominant source of Th2 cytokines [41]. Meanwhile, multiple studies have corroborated a critical role for CCR4 in innate immune cell activation and Th2-associated immunopathologies [11,42,43].

CCR4 has also been shown to play a detrimental role in septic shock. *Ccr4* knockout mice showed enhanced survival in lipopolysaccharide-induced endotoxic shock that was lethal to wild-type littermates. The survival of these animals was associated with lower serum levels of pro-inflammatory cytokines and a decreased influx of macrophages into the peritoneal cavity [35]. Likewise, *Ccr4*-deficient mice show improved survival rates in the colon ascendens stent peritonitis model of polymicrobial abdominal sepsis [44]. The observation of features associated with alternative macrophage activation among *Ccr4*-deficient macrophages may provide important clues as to the mechanism underlying these findings [43].

### 3.3. Identification and Cellular Expression of CCL17

In 1996, a gene coding for a novel C-C chemokine, originally known as thymus and activation-regulated chemokine (TARC), was cloned and later designated C-C chemokine ligand 17 (CCL17). CCL17 mRNA was shown to be expressed constitutively in thymus tissue and transiently in stimulated peripheral blood mononuclear cells. CCL17 induced chemotaxis and Ca^2+^ influx in the cells stably expressing CCR4, thereby demonstrating the ligand–receptor relationship between CCL17 and CCR4 [45]. Interestingly, in a differential mRNA display study designed to compare the gene expression profiles of DCs and macrophages, Lieberam and Förster (1999) found that CCL17 mRNA was expressed specifically in a subset of murine bone-marrow-derived DCs [46]. The subsequent generation of *Ccl17* mutant animals greatly helped to unravel the role of CCL17 in various infectious and autoimmune disorders [47,48,49,50].

Using a CCL17/enhanced green fluorescent protein (EGFP) reporter mouse model to identify cellular sources of CCL17 in vivo, we demonstrated that DCs are an important cellular source for CCL17 during both homeostasis and inflammation [47]. Two major subsets of DCs have been classified on the basis of functional and morphological characteristics, namely conventional or classical DCs (cDCs) and plasmacytoid DCs (pDCs). cDCs express high levels of major histocompatibility complex class II molecules and exert potent phagocytotic and antigen presentation capacities. cDCs can be subdivided based on surface marker expression, CD11b^+^ cDCs, which activate CD4^+^ T cells preferentially, and CD8α^+^ cDCs, which are highly efficient cross-presenting cells [51]. Alternatively, DCs have also been divided into DC1 and DC2 subpopulations based on their capacity to induce Th1 and Th2 cell differentiation in vitro, respectively [52]. Utilizing the aforementioned CCL17 reporter mouse model, we found that CCL17 was produced mainly by a subset of CD11b^+^ cDCs located in primary and secondary lymphoid organs, but not the spleen. Toll-like receptor stimulation upregulated CCL17 expression in CD11b^+^ cDCs in lymph nodes, but did not induce CD11b^+^ cDCs in the spleen to express CCL17 [47]. To unravel these surprising results, Globisch et al. performed a genome-wide expression profiling, which demonstrated that IFN-γ suppresses CCL17 production by CD11b^+^ DCs in the spleen. Meanwhile, CCL17-producing CD11b^+^ DCs in the lymph nodes had low responsivity to IFN-γ due to downregulation of IFN-γ receptors [48]. The findings of this study underscored that the cytokine milieu and differential responsiveness of DC subsets controls the organ/tissue-specific immune status represented by the chemokine expression pattern. The plasticity of this expression was demonstrated by a further study, wherein NK T cell activation in mice after the systemic application of α-galactosylceramide “licensed” CD8α^+^ DCs in the spleen for crosspriming, and thus was able to release the CCL17 production block in these cells [53].

### 3.4. Involvement of CCL17 in Various Diseases

CCL17 is involved in the induction or enhancement of a broad spectrum of immune reactions, ranging from contact hypersensitivity responses and allograft rejection to inflammatory bowel disease and various inflammatory diseases, such as atopic dermatitis and atherosclerosis [47,49,50,54]. Several CCL17- and CCR4-mediated mechanisms have been postulated. The reduction of atherosclerosis in *Ccl17*-deficient atherosclerosis-prone mice was shown to be dependent on Treg cells, and CCL17 expression by DCs limited the expansion of that cell subset [54]. Likewise, in intestinal inflammation in mice, CCL17 reduced Treg cell expansion, but promoted IL-12 and IL-23 production by DCs in an autocrine mode and augmented activation of Th1 and Th17 cells [49].

### 3.5. Identification and Cellular Expression of the Second CCR4 Ligand CCL22

The second CCR4 ligand, the C-C chemokine ligand CCL22, originally known as macrophage-derived chemokine, shares 37% identity at the amino acid level with CCL17 [55]. The genes that encode CCL17 and CCL22 are both in close proximity to the CX3CL1-encoding gene on human chromosome 16q13 [56]. Like CCL17, CCL22 is highly expressed in the thymus and by myeloid cells. Alternatively activated (M2) macrophages typically associated with Th2 responses and tissue repair have been shown to produce CCL22 in high amounts [57]. Monocyte-derived DCs have also been reported to produce CCL22, thereby leading to the rapid binding of DCs to activated T cells under dynamic conditions. These findings underscored that chemokine-dependent binding of activated T cells to DCs represents an important step in T cell priming [58]. Microbial products, such as lipopolysaccharide, and cytokines, such as IL-1β, tumor necrosis factor (TNF), and CD40 ligand, have been shown to induce CCL22 production in DCs [59]. Interestingly, supernatants from stimulated DCs were found to contain the full-length molecule as well as N-terminally truncated shorter forms of CCL22 which do not recognize CCR4. It has been suggested that the expression of these truncated peptides may represent a maturation-dependent negative feedback mechanism in DC-mediated immune responses [60]. 

Functionally, CCL22 is a potent chemoattractant for antigen-experienced, but not resting, T lymphocytes, as well as for NK cells, monocytes, and DCs [59]. CCL22 production in myeloid cells can be induced by the Th2 cytokines IL-4 and IL-13 and is inhibited by the Th1 cytokine IFN-γ, which may enable an amplification loop for polarized Th2 responses [59,61]. However, IL-4 and IL-13 have also been shown to down-regulate TNF and IFN-γ enhanced CCL22 production in keratinocytes, suggesting cell-specific regulation of CCL22 [62].

### 3.6. Involvement of CCL22 in Various Diseases

CCL22 is thought to be involved in diverse pathologies, ranging from allergic reactions and autoimmunity to tumor growth. Increased CCL22 expression has been observed in allergy and inflammatory skin responses [59]. In the lung, DC and alveolar macrophage depletion protected mice from IL-13-induced airway inflammation, and this protection was associated with reduced CCL17 and CCL22 production [63]. Interestingly, the immunomodulatory properties of CCL22 in autoimmunity involve Treg cell responses. Overexpression of CCL22 in pancreatic β cells in type 1 diabetes model mice prevented autoimmune attack by recruiting Treg cells, ultimately providing protection from diabetes progression [64]. CCL22 mediated control of Treg cell biology has also been evidenced in various human tumors. Tumor cells and microenvironmental macrophages produce CCL22, thereby recruiting Treg cells and suppressing tumor-specific T cell immunity. This detrimental action of CCL22 may represent a tumor immune-escape response mechanism [65]. In vivo CCL22 deficiency studies are urgently needed to elucidate CCL22 biology in relation to infection, autoimmunity, and cancer.

### 3.7. Known and Putative Ligands for CCR4

Both CCL22 and CCL17 are robust ligands for CCR4 [28]. In this context of dual chemokines potentially competing for the same receptor, N-terminal processing represents a mechanism of the fine-tuning of chemokine activity that can render particular chemokines inactive or antagonistic. In CCL22, N-terminal truncations have been shown to confer a loss of chemoattractant activity for T-cells, but the retention of chemotactic capacity for monocytes. Based on these results, the existence of an alternative receptor for CCL22 that may be differentially stimulated by the two CCL22 forms was postulated [66]. As expected, splenocytes from *Ccr4*-knockout mice were unresponsive to CCL17 and CCL22. Interestingly, at high doses, CCL2, CCL3, and CCL5 induced signaling via CCR4 [26] and *Ccr4*-deficient cells did not show a chemotactic response to CCL3 in vitro [35]. These findings suggested that CCL3 could also exert chemotactic effects via CCR4, an unresolved issue until now. 

In the next chapter, we will focus on the role of CCR4 and its ligands in the pathogenesis of CNS autoimmunity. 

## 4. CCR4, CCL17, and CCL22 in Multiple Sclerosis (MS)

### 4.1. Involvement of the CCR4-CCL17/CCL22 Axis in the Pathogenesis of MS

There is growing clinical evidence suggestive of functional involvement of the CCR4-CCL17/CCL22 axis in the pathogenesis of MS. A study analyzing the allelic frequency of single nucleotide polymorphisms (SNPs) in the coding sequences of CCL17 and CCL22 in MS patients, versus in healthy controls, indicated an association of SNPs in this chemokine cluster with MS risk, particularly in males [67]. Cerebrospinal fluid (CSF) levels of CCL22 and CCL17 have been reported to be elevated in MS patients [68,69]. In a cross-sectional study, increased levels of CCL22, CXCL10, and sCD40L in the CSF characterized relapsing-remitting MS patients with the presence of gadolinium-enhancing lesions [70]. One study reported increased CCL22 levels exclusively in female MS patients, relative to both female controls and male MS patients, suggesting a potential pathogenic role of CCL22 exclusively in females with MS [67].

The cellular expression pattern of CCR4 and its ligands in human CNS has also been investigated and is summarized in Table 1. Direct data from the CNS of MS patients, however, is relatively scarce. No expression of CCR4 or CCL17 was found on leukocytes in brain sections from patients with active as well as inactive MS [71]. Neuropathological studies of postmortem CNS samples demonstrated that activated microglia in lesions in both normal-appearing white matter (inactive or potentially preactive lesions) [72] and remyelinated areas express CCL22, an M2 marker, as well as M1 markers (e.g., CD40 and CD86). These data suggest that there are heterogeneous microglia clusters or a potential M1/M2 phenotypic switch in microglia in MS lesions [73]. In vitro, microglia were also shown to upregulate CCL17 expression after stimulation with 1,25-Dihydroxyvitamin D3 [74] and to exhibit low-level basal expression of CCR4, as did primary astrocytes [75].

Additionally, circulating NK cells that exhibit cytolytic and immunoregulatory functions in the CSF of MS patients were shown to produce CCL22 in response to IL-2 activation in vitro [76]. The respective contribution of different CNS resident and infiltrating cell types to the CCR4-, CCL17- and CCL22-mediated effects in the course of MS disease awaits further clarification.

### 4.2. Expression of CCR4, CCL17, and CCL22 in MS Treatment

Clinical studies have examined how MS therapy affects the expression of CCL17/CCL22 and CCR4. Treatment with natalizumab, a blocking antibody against the adhesion molecule integrin α4 and thereby a putative inhibitor of leukocyte transmigration to the CNS [77,78], was found to be associated with reduced CSF levels of CCL22, while CCL17 was below the detection threshold in MS patients before and after natalizumab treatment. Meanwhile, the circulating plasma levels of granulocyte-macrophage colony-stimulating factor (GM-CSF), an important inductor cytokine for CCL17 and CCL22 in DCs [37], also decreased after one year of treatment. This indicates a global decline in cytokine and chemokine levels and an additional immunoregulatory capacity of natalizumab apart from inhibiting transmigration [79]. Similarly, intravenous methylprednisolone therapy has been reported to reduce CSF concentrations of CCL17 and IL-17 in relapsing-remitting MS patients [80]. IFN-β therapy suppresses MS disease activity [81] and high-dose IFN-β formulations have been shown to suppress CCL17 levels in peripheral blood [82]. These findings suggest that CCL17 and CCL22 concentrations in the CSF of patients with MS may be influenced by treatments. 

On the receptor side, many studies have demonstrated that CCR4 transcripts and CCR4-expressing CD4^+^ T cells in the CNS or blood are abnormally low in patients with active MS. For example, CD4^+^ and CD8^+^ T cells in CSF during MS relapse had enriched CXCR3 expression, but reduced CCR3 and CCR4 expression, compared with CD4^+^ and CD8^+^ T cells in the blood [83]. An increased ratio of CXCR3^+^/CCR4^+^ CD4^+^ T cells in the blood during relapse, compared to during remission, has since been suggested to be a correlate of disease activity [84]. Conversely, Matsui et al. postulated that IL-4-producing, CCR4-expressing Th cells were persistently decreased in the blood of MS patients, relative to healthy controls, independent of MS stage [85]. With regard to treatment, IFN-β treatment has been shown to increase CD4^+^ T-cell surface expression of CCR4, while the expression of CCR4 was significantly lower in untreated MS patients compared with healthy volunteers [86]. Furthermore, the treatment of secondary progressive MS with cyclophosphamide, which has both immunosuppressive and immunomodulatory effects, increased the percentages of CCR4-expressing T cells that produce IL-4 and reversed detrimental IFN-γ production in CD8^+^ T cells [87]. An exception provides high-dose intravenous methylprednisolone treatment in MS patients, that has been shown to reduce the portions CD4^+^ T cells expressing CD25 and CCR4 in the CSF within two weeks of treatment initiation [88]. In a functional study, Høglund et al. (2011) characterized the expression and activity of chemokine receptors in glatiramer acetate (GA)-specific T cells isolated from MS patients receiving a GA treatment. They found that GA-reactive CD4^+^ T cell clones from blood and CSF shared expression of several receptors, including CCR4, and showed chemotaxis toward CCL22, suggesting that CCR4 may be involved in T cell migration from blood to CSF in MS [89]. Taken together, these heterogenic findings underscore the need for large cohort studies to clarify the specific roles of CCL17, CCL22, and CCR4 in MS disease development and treatment–response processes. 

## 5. CCR4-, CCL17-, and CCL22-Mediated Mechanisms in Experimental Autoimmune Encephalomyelitis (EAE)

### 5.1. Expression of CCR4, CCL17, and CCL22 in EAE

EAE animal models of MS have provided critical information about the mechanistic contributions of CCL17, CCL22, and CCR4 to the pathogenesis of CNS autoimmunity. Elevated *Ccl22* and *Ccr4* mRNAs have been detected in the CNS of mice developing the relapsing-remitting and chronic-relapsing forms of EAE induced by proteolipid peptide (PLP)_139–151_. Both transcripts were found in CNS-infiltrating leukocytes, while only *Ccl22* was found in microglia. Upregulation of CCL22 in activated microglia was shown to induce Th2 lymphocyte chemotaxis in vitro, suggesting that microglia play a role in Th2-cell recruitment to inflammatory sites [36]. In this model, CCR4 expression by T cells may be restricted to CNS-immigrating encephalitogenic T cells [90]; CCR4 was not detectable in resting and lymph-node-derived T cells from PLP_139–151_ immunized Swiss Jim Lambert SJL mice. Conversely, PLP_139–151_ re-stimulation of primed CD4 T cells in vitro resulted in CCR4 upregulation. 

### 5.2. Regulation of CCR4, CCL17, and CCL22 Expression during Treatment Approaches in EAE

It is noteworthy that in analogy to MS, beneficial therapeutic outcomes in EAE have been associated with increased levels of CCL17/CCL22-CCR4 axis components. For example, IL4 gene therapy, which ameliorates EAE neuropathology, has been shown to enhance production of CCL1, CCL17, and CCL22, capable of recruiting protective Treg cells [91]. The copolymer glatiramer acetate (Y, E, A, K)_n_ called YEAK has MS treatment efficacy; analogous amelioration of EAE with the second generation copolymer (Y, F, A, K)_n_ called YFAK is associated with induction of IL-10-secreting Treg cells and M2 regulatory macrophages. Administration of YFAK or YEAK raises plasma CCL22 levels in mice, and bone-marrow-derived macrophages secrete CCL22 in vitro in response to YFAK and, at higher concentrations, also to YEAK [92]. In transgenic mice expressing a BV8S2 T cell receptor specific for myelin basic protein-NAc1–11 peptide, vaccination with BV8S2 protein was found to be protective against EAE, to augment the differentiation of a subset of BV8S2-specific Treg cells, and to increase the CNS expression of CCR3 and CCR4 [93]. Similarly, altered peptide ligand-induced protection from EAE in SJL mice was accompanied by an increase in spinal cord CCR3 and CCR4 expression, without detectable changes in Th1 associated chemokine receptors [94]. In a marmoset EAE model, intranasal application of a tolerogenic myelin oligodendrocyte glycoprotein (MOG)_10–60_ containing fusion-protein-induced immunomodulation was characterized by increased IL-4 and IL-10 production and increased CCR4 expression in the spleen, but did not affect disease severity [95]. Although these studies pointed toward a link between protection from EAE and increased levels of CCR4, IFN-β treatment in the mice, which inhibited EAE severity, neuroinflammation, and demyelination, was associated with reduced CNS levels of various chemokine receptors, including CCR4 and its corresponding ligand CCL22 [96]. In another report, mice with T-cell-specific deletion of the histone deacetylase 1 gene, which confers resistance to EAE, exhibited T-cell-specific increases in phosphorylated STAT1 levels and reduced expression of CCR4 and CCR6 [97].

### 5.3. EAE Induction in Gene Mutant Mice

Interestingly, studies of *Ccr4-* and *Ccl17*-deficient mice have been suggestive of a disease-promoting influence of this chemokine/receptor-axis. *Ccr4*-deficient mice that exhibited reduced EAE incidence and severity had reduced numbers of TNF-producing inflammatory Ly6C^hi^ CD11b^+^ cells in the periphery and spinal cord [98]. A study examining *Ccr4*^−/−^ and/or *Ccr6*^−/−^ knockout mice subjected to MOG immunization showed that although deletion of either gene alone did not alter the disease course, double knockout mice developed less severe EAE and presented reduced Th17 recall responses, relative to control mice [99]. 

Our own studies have provided evidence indicating that CCR4 and CCL17 modulate EAE pathogeneses by affecting the basic functions of DCs. We found that DC-specific expression of CCR4 is required for the development of EAE. *Ccr4*^−/−^ animals were fully resistant to MOG-induced EAE and showed reduced neuroinflammation and demyelination. *Ccr4*-deficient DCs exhibited diminished production of GM-CSF and IL-23, and they showed a diminished Th17 response maintenance capacity in vitro. EAE susceptibility has been restored to *Ccr4*^−/−^ mice by intracerebral microinjection of CCR4^+^ DCs. This prominent role of CCR4-expressing DCs in EAE is somewhat surprising given that DCs express far lower levels of CCR4 than T-cell subsets [37].

In a follow-up study, we investigated the expression pattern and functional role of CCL17 in EAE. MOG-immunization of CCL17/EGFP reporter mice revealed that CCL17 was expressed in a subset of cDCs that migrated into the CNS during the effector phase of EAE. *Ccl17*-deficient mice showed an ameliorated disease course after MOG-immunization, and this amelioration was associated with the reduced migration of IL-17-producing CD4^+^ T cells and peripheral DCs into the CNS. These animals also exhibited elevated levels of splenic Treg cells at peak disease. Additionally, *Ccl17*-deficient DCs showed reduced transmigration across an in vitro blood–brain barrier model. These findings showed that CCL17 modulates EAE pathogenesis by regulating DC trafficking as well as by expanding peripheral Treg cell populations [100]. With regard to CCL22 involvement in EAE, until now, a neutralizing antibody treatment approach has been performed. Neutralizing CCL22 activity during EAE at the time of myelin antigen immunization resulted in reduced clinical severity and demyelination, associated with lower numbers of CD11b^+^ Ly6C^hi^ cells in the CNS when compared to control antibody-treated mice [101]. Future generation of *Ccl22* knockout mice is essential for understanding the role of this chemokine in CNS autoimmunity.

Altogether, the findings reviewed here demonstrate that CCR4 and CCL17 operate at distinct levels and on different cell subsets during the EAE-associated immune response (Table 2). Thus, CCR4 may act through a variety of functionally distinct cell types, some of which may have opposing activities. This complexity of CCR4-mediated effects in CNS autoimmunity pathogenesis may underlie the discrepancies in findings regarding CCR4 alterations in therapy studies versus gene-deficient animal studies. A deeper understanding of the key role of CCR4 and its ligands in EAE may enable novel strategies for the pharmacological targeting of CCR4 on DCs to be developed for inhibiting destructive CNS autoimmunity. 

## 6. Therapeutic Targeting of CCL17/CCL22 and CCR4

### 6.1. Differences in CCL17- and CCL22-Mediated Studies in Gene Mutant Mice

As the biology of the CCL17/CCL22-CCR4 chemokine–receptor axis is being further elucidated, interest in its constituent molecules as potential therapeutic targets for the treatment of allergies, autoimmune diseases, and cancer has been growing. Small molecule compounds and antibodies with the capacity to block CCL17-and CCL22-mediated recruitment of Th2 cells and Treg cells have been shown to produce positive effects in various disease models for asthma, atopic diseases, and tumor growth [28]. Strategies to develop anti-CCR4 compounds have been reviewed before [28,102,103]. Here, we highlight recent selected approaches aimed at blocking CCR4-mediated actions.

As chemokine receptor internalization may be induced upon ligand binding, the removal of a chemokine receptor’s cognate ligand may lead to a resurgence of receptor surface expression levels, and may thereby restore the chemotactic sensitivity of CCR4-expressing cells. CCL22 is a potent inducer of CCR4 internalization, and CCL22 binding of CCR4 can reduce the subsequent functional responsiveness of CCR4. CCL17, by comparison, is a weaker inducer of CCR4 internalization [104]. Additionally, relative to CCL17, CCL22 mediates more efficient β-arrestin recruitment via CCR4, which is important for regulating signal transduction. These differences between CCL17 and CCL22 effects may provide an opportunity for the selective modulation of CCR4 signaling in immune cell trafficking and responses. 

### 6.2. Small-Molecule Competitive Antagonists against CCR4

Multiple small-molecule competitive antagonists that prevent CCL17 and CCL22 from triggering the CCR4-mediated chemotaxis of CCR4-expressing cells have been developed [105]. Purandare and Somerville (2006) described four groups of CCR4 antagonists based on their chemical characteristics: aryl sulphonamides, substituted amino heterocycles, thiazolidinones, and lactams [106]. Alternatively, CCR4 antagonists have been grouped into the two categories of lipophilic heteroarenes and aryl sulphonamides [103]. Several small-molecule CCR4 antagonists have been shown to have efficacy in allergy and tumor growth disease models, and have thus been advanced to a clinical study stage. However, most CCR antagonists that have been tested have failed to be approved for disease treatment. This may be caused by the yet unresolved complex pharmacology involving the regulation of CCR4 surface expression and trafficking [107]. Like endogenous ligands, small-molecule antagonists also show differential capacities for inducing receptor internalization, and this variation may be associated with differing biological efficacies. Some CCR4 antagonists bind allosteric sites on CCR4 that do not overlap with ligand binding sites [108]. Of note, Ajram et al. described antagonist binding at an intracellular allosteric site on CCR4 that appeared to evoke receptor internalization [109]. Only one CCR4 antagonist, indazole arylsulfonamide GSK 2239633, has been submitted in phase I clinical trials as a possible treatment for asthma, but it was not developed further due to unresolved challenges, including low bioavailability [105].

### 6.3. Humanized Anti-CCR4 Antibody and Neutraligands 

Several groups have developed antagonistic monoclonal antibodies against CCR4 [103,110]. The humanized anti-CCR4 antibody mogamulizumab has demonstrated a high potential for treatment of CCR4-positive T-cell lymphomas [111]. Mogamulizumab enhances antibody-dependent cellular cytotoxicity markedly through reduced fucosylation of the Fc region of antibodies and was approved in Japan for the treatment of patients with relapsed/refractory CCR4-positive adult T-cell lymphoma [112]. Furthermore, a multi-center phase III study of mogamulizumab versus vorinostat, a histone deacetylase inhibitor, has been initiated in patients with CCR4-positive cutaneous T-cell lymphoma. Finally, a multi-center phase Ib trial examining a combination treatment consisting of mogamulizumab and utomilumab, a 4-1BB agonist, for the treatment of advanced solid tumors was started (ClinicalTrials.gov Identifier: NCT02444793) [113]. Mechanistically, mogamulizumab is thought to also target CCR4-positive Treg cells and thereby facilitate antitumor immunity. However, this inhibition of Treg cellular functions can result in severe adverse effects affecting the skin, underscoring the inherent risks of altering Treg cell biology [113]. Taking a ligand-sided approach, Abboud and colleagues (2015) have identified decoy molecules for CCL17 and CCL22, termed neutraligands, with anti-inflammatory activity. In vitro experiments have indicated that these neutraligands can inhibit CCL17- or CCL22-induced intracellular calcium responses, CCR4 endocytosis, and human T cell migration. Furthermore, in an in vivo experiment, neutraligands inhibited inflammation in a murine model of asthma [114].

### 6.4. Anti-CCR4 Antagonist Studies in EAE

There has been limited research that has examined CCR4 antagonist effects on CNS autoimmunity. One CCR4 antagonist, compound 22, was reported to ameliorate EAE, suggesting that CCR4 antagonism may be a potential therapeutic strategy for MS [115]. Conversely, the CCR4 antagonist AF399/420/18025 did not have a significant effect on the EAE clinical score [116]. It is possible that the use of dimethyl sulfoxide as a drug vehicle solution for CCR4 antagonists may affect the blood–brain barrier and thus influence infiltration of mononuclear cells into the CNS [117]. Future studies are needed to evaluate whether any of the aforementioned anti-CCL17/CCL22-CCR4 chemokine–receptor axis agents discussed here may be useful for CNS autoimmunity therapy.

## 7. Conclusions

The CCL17/CCL22-CCR4 chemokine–receptor axis represents a promising assemblage of potential novel targets for the treatment and prevention of CNS autoimmune diseases. The great complexity of CCR4 regulation and activities in the context of CNS autoimmune diseases may explain, at least in part, discrepant findings between studies utilizing mutant mice and pharmacological approaches. Our studies with *Ccr4*- and *Ccl17*-deficient mice pointed toward a prominent modulatory effect of CCL17-CCR4 signaling on DC-mediated functions in EAE, though CCR4 is only minimally expressed on DCs. A deeper understanding of the central role of CCR4 and its ligands on DCs in EAE may help us to exploit CCR4 modulation as a strategy for inhibiting CNS autoimmunity by targeting DC-expressed CCR4. The clinical utility of antagonizing compounds, mogamulizumab and neutraligands, for the treatment of CNS autoimmunity remains to be clarified. In this regard, the hierarchy of CCR4-mediated actions on the diverse players in CNS autoimmunity that could be modulated by anti-CCR4 compounds needs to be determined. Future prospects for CCR4 targeting include a structure-based design that takes into account allosteric binding sites and the differential modulation of receptor trafficking by diverse antagonists. 

Although the findings of animal model studies have been encouraging, there are many challenges that still need to be resolved before CCR4-targeted therapies for CNS autoimmunity can be successfully developed for patients.

## Figures and Tables

**Table 1:** Cellular expression of CCR4, CCL17, and CCL22 in the human CNS.

Cell Type	Chemokine/Chemokine Receptor Investigated	Description/Inflammatory Context	References
CNS infiltrating T cells	CCR4 and CCL17	No expression detected in leukocytes in brain sections from MS patients	[71]
Microglia	CCL22	Activated microglia in lesions and remyelinated areas	[72,73]
Microglia	CCL17	The immortalized human microglial cell line HMO6 upregulated CCL17 after stimulation with 1,25-Dihydroxyvitamin D3 in vitro	[74]
Microglia and astrocytes	CCR4	The human microglial cell line CHME3 and primary astrocytes express low levels of CCR4 mRNA	[75]

CCR4: chemokine receptor 4; CCL17: C-C chemokine ligand 17; CCL22: C-C chemokine ligand 22; CNS: central nervous system.

**Table 2:** Genetic and functional ablation of CCL17/CCL22 and CCR4 in EAE.

Gene Mutant/Pharmacological Blockade	Clincical Score of EAE	Proposed Mechanism	References
*Ccr4*^−/−^	Reduced	Reduced numbers of tumor necrosis factor-producing inflammatory Ly6C^hi^ CD11b^+^ cells in the periphery and spinal cord	[98]
*Ccr4*^−/−^	Markedly reduced	Reduced capacity of DCs to produce IL-23 and GM-CSF and maintain survival of Th17 cells	[37]
*Ccr4*^−/−^ × *Ccr6*^−/−^	Reduced	Reduced Th17 recall responses	[99]
*Ccl17*^E/E 1^	Mildly reduced	Reduced immigration of DCs into the CNS and diminished transmigration capacity across a blood-brain barrier model, accumulation of splenic T_reg_ cells	[100]
Anti-CCL22 antibody-mediated blockade	Reduced	Lower numbers of CD11b^+^ Ly6C^hi^ cells in the CNS	[101]

DC: dendritic cell; CNS: central nervous system; EAE: Experimental autoimmune encephalomyelitis; GM-CSF: granulocyte-macrophage colony-stimulating factor; ^1^ *Ccl17*^E/E^ mice are homozygous for a targeted insertion of an EGFP reporter gene into the *Ccl17* locus and thus are CCL17 deficient [47].

## Abbreviations

CNS	Central nervous system
CCL17	C-C chemokine ligand CCL17
CCL22	C-C chemokine ligand CCL22
CCR4	C-C chemokine receptor 4
EAE	Experimental autoimmune encephalomyelitis
GM-CSF	Granulocyte-macrophage colony-stimulating factor
IL	Interleukin
MOG	Myelin oligodendrocyte glycoprotein
